# 84. Paying for Parenthood: Misinterpretation of ABIM Leave Policies May Lead to Unnecessary Extension of ID Fellowships

**DOI:** 10.1093/ofid/ofab466.084

**Published:** 2021-12-04

**Authors:** Wendy Stead, Catherine P Gardiner, Laura P Desrochers, Kathleen Finn, Furman S McDonald, Michael Melfe, Michael Melia

**Affiliations:** 1 Beth Israel Deaconess Medical Center, Boston, Massachusetts; 2 Massachusetts General Hospital/Harvard Medical School, Boston, Massachusetts; 3 American Board of Internal Medicine, Philadelphia, Pennsylvania; 4 Johns Hopkins University, Baltimore, Maryland

## Abstract

**Background:**

Many trainees plan pregnancy during fellowship training. A study of internal medicine program directors (PDs) demonstrated frequent misinterpretation of American Board of Internal Medicine (ABIM) leave policies when applied to parental leave. The ABIM has since attempted to clarify its leave and deficits in training policies. The primary aim of this study was to investigate how infectious disease (ID) program directors interpret the current ABIM leave policies in crafting parental leave for trainees.

**Methods:**

We surveyed 155 ID program directors in an online, anonymous questionnaire regarding their knowledge of ABIM leave policies and application toward trainees’ leaves of absence.

**Results:**

75/155 (48%) of program directors responded to the survey. Most respondents incorrectly identified the leave limits permitted by ABIM policies, and a majority mistakenly chose to extend training when a clinically competent fellow was within their allowed duration of leave.(Figure 1) Most respondents correctly identified that equal time is permitted for both birth and non-birth parent parental leave, however, reported leave durations did not reflect this equity. PDs reported the majority (60.4%) of ID trainee maternity/birth parent leaves at their programs were ≤7weeks and 4.6% were≤3 weeks, while only 7% were≥12 weeks. In contrast, 50% of paternity/non birth parent leaves were ≤3weeks and none were ≥12 weeks. (Figure 2) PDs utilize various strategies to prevent extending training for fellows taking parental leaves that exceed the limits allowed by ABIM policies, including creating “home electives,” though 34% counsel trainees to take “a shorter maternity leave.”

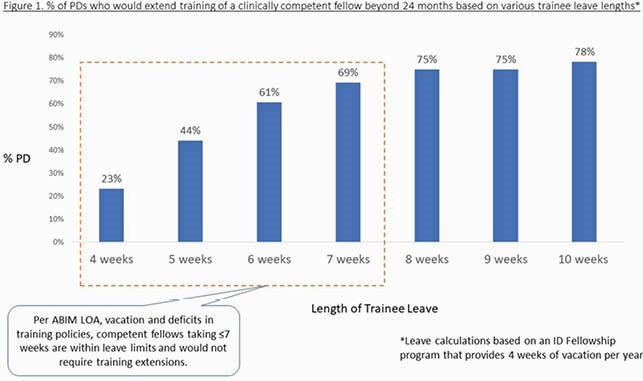

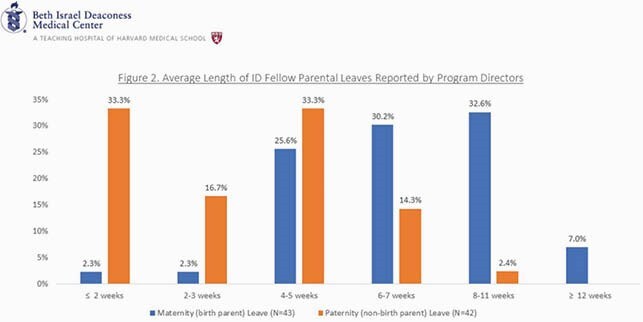

**Conclusion:**

Fellowship program directors often misinterpret ABIM leave policies, and misapply them when given example scenarios. These findings have clear implications for trainees’ family planning and may lead to shortened parental leaves and inappropriate fellowship training extensions.

**Disclosures:**

**All Authors**: No reported disclosures

